# Mitochondrial DNA leakage induces odontoblast inflammation via the cGAS-STING pathway

**DOI:** 10.1186/s12964-021-00738-7

**Published:** 2021-05-20

**Authors:** Lu Zhou, Yi-Fei Zhang, Fu-Hua Yang, Han-Qing Mao, Zhi Chen, Lu Zhang

**Affiliations:** 1 The State Key Laboratory Breeding Base of Basic Science of Stomatology (Hubei- MOST) and Key Laboratory of Oral Biomedicine Ministry of Education, School and Hospital of Stomatology, Wuhan University, Wuhan, China; 2 Department of Endodontics, School and Hospital of Stomatology, Wuhan University, HongShan District, LuoYu Road No. 237, Wuhan, 430079 China

**Keywords:** Mitochondrial DNA, Mitochondrial damage, cGAS-STING pathway, Odontoblasts, Inflammation, Immune response

## Abstract

**Background:**

Mitochondrial DNA (mtDNA) is a vital driver of inflammation when it leaks from damaged mitochondria into the cytosol. mtDNA stress may contribute to cyclic GMP-AMP synthase (cGAS) stimulator of interferon genes (STING) pathway activation in infectious diseases. Odontoblasts are the first cells challenged by cariogenic bacteria and involved in maintenance of the pulp immune and inflammatory responses to dentine-invading pathogens. In this study, we investigated that mtDNA as an important inflammatory driver participated in defending against bacterial invasion via cGAS-STING pathway in odontoblasts.

**Methods:**

The normal tissues, caries tissues and pulpitis tissues were measured by western blotting and immunohistochemical staining. Pulpitis model was built in vitro to evaluated the effect of the cGAS-STING pathway in odontoblast-like cell line (mDPC6T) under inflammation. Western blot and real-time PCR were performed to detect the expression of cGAS-STING pathway and pro-inflammatory cytokines. The mitochondrial function was evaluated reactive oxygen species (ROS) generated by mitochondria using MitoSOX Red dye staining. Cytosolic DNA was assessed by immunofluorescent staining and real-time PCR in mDPC6T cells after LPS stimulation. Furthermore, mDPC6T cells were treated with ethidium bromide (EtBr) to deplete mtDNA or transfected with isolated mtDNA. The expression of cGAS-STING pathway and pro-inflammatory cytokines were measured.

**Results:**

The high expression of cGAS and STING in caries and pulpitis tissues in patients, which was associated with inflammatory progression. The cGAS-STING pathway was activated in inflamed mDPC6T. STING knockdown inhibited the nuclear import of p65 and IRF3 and restricted the secretion of the inflammatory cytokines CXCL10 and IL-6 induced by LPS. LPS caused mitochondrial damage in mDPC6T, which promoted mtDNA leakage into the cytosol. Depletion of mtDNA inhibited the cGAS-STING pathway and nuclear translocation of p65 and IRF3. Moreover, repletion of mtDNA rescued the inflammatory response, which was inhibited by STING knockdown.

**Conclusion:**

Our study systematically identified a novel mechanism of LPS-induced odontoblast inflammation, which involved mtDNA leakage from damaged mitochondria into the cytosol stimulating the cGAS-STING pathway and the inflammatory cytokines IL-6 and CXCL10 secretion. The mtDNA-cGAS-STING axis could be a potent therapeutic target to prevent severe bacterial inflammation in pulpitis.

**Video Abstract**

**Graphic abstract:**

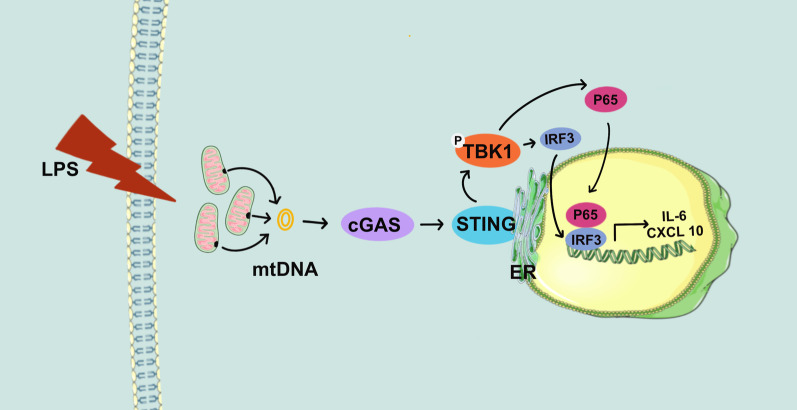

**Supplementary Information:**

The online version contains supplementary material available at 10.1186/s12964-021-00738-7.

## Background

According to Global Burden of Diseases, Injuries, and Risk Factors Study 2016 data, the greatest prevalence was dental caries among all disease and its global incidence was the second [1]. Dental caries is a bacterial infectious disease in hard tissue, which lead to tooth defects and even pulpitis, causing severe pain and seriously affecting the quality of life of patients. Odontoblasts play a crucial role in maintaining a balanced pulp microenvironment to regulate transcellular transport between the pulp and dentine [2]. During the progression of caries, odontoblasts can recognize and respond to bacteria, and these cells are the first line of defense against cariogenic bacteria for their specific localization at the pulp-dentin interface [3]. Thus, it is of importance to elucidate the mechanisms by which odontoblasts eliminate invading bacteria.

The initial function of the cGAS-STING pathway is host defense, but recent studies have revealed its fundamental roles in the development of a variety of inflammatory diseases [4–6]. Cyclic GMP-AMP (cGAMP) synthase (cGAS) can detect intracellular DNA and generate the second messenger cGAMP, thereby activating STING in the endoplasmic reticulum [7]. Then, activated STING is transported from the ER to the Golgi, where it forms a complex. This complex with TANK-binding kinase 1 (TBK1) is then transferred to the endolysosome, in which it stimulates transcription factors such as nuclear factor kappa-B (NF-κB) and interferon regulatory factor 3 (IRF3). These transcription factors facilitate the expression of type I interferons or proinflammatory cytokines [8–10]. Emerging evidence shows that the cGAS-STING pathway is activated not only by nonself DNA, such as DNA from viruses, bacteria, and protozoa but also by self-DNA, including intracellular, mitochondrial and nuclear DNA, which can enter the cytoplasm [6].

Mitochondrial DNA (mtDNA), which is the only nonnuclear genome, is double-stranded circular DNA (16.5 kbp), is a component of mitochondria [11]. mtDNA encodes 37 genes, all of which are associated with oxidative phosphorylation and normal mitochondrial function [12]. Compared with nuclear DNA (nDNA), mtDNA is more unstable and vulnerable to the effects of oxidative stress due to its close proximity to mitochondrial reactive oxygen species (mtROS) and a lack of repair machinery [13, 14]. Accumulating evidence indicates that when mtDNA leaks from damaged mitochondria into the cytosol, it becomes a vital driver of inflammation [11, 15, 16]. mtDNA serves as a kind of mitochondrial danger-associated molecular patterns (mtDAMPs) that can engage various pattern recognition receptors (PRRs) and activate the innate immune system, including cGAS-STING [15, 17, 18].

mtDNA stress may contribute to cGAS-STING pathway activation and type I IFN responses in various pathological states, including infectious diseases, cancer, neurodegeneration and other mitochondria-related illnesses [15, 16]. Systemic lupus erythematosus (SLE) neutrophils release mtDNA, which activates the cGAS-STING pathway to promote type I IFN-regulated autoimmunity [19]. Microorganisms such as *Mycobacterium tuberculosis* can stimulate cGAS activation for mitochondrial damage and mtDNA release, which enhances intracellular survival [20]. Beyond stimulating T cell proliferation, activation of the cGAS-STING pathway causes tumor vascular collapse and contributes to tumor cell death and apoptosis, promoting the release of tumor-associated antigens [6]. Despite numerous studies describing the importance of the cGAS-STING pathway in various pathological processes, it is still unclear whether the cGAS-STING pathway is involved in inflamed odontoblasts.

The present study identified a distinct mechanism of LPS-induced odontoblast inflammation, which involved the cGAS-STING pathway. Mitochondrial dysfunction and mtDNA leakage into the cytosol were induced by LPS via the cGAS-STING pathway, leading to odontoblast-like cell inflammation. The mtDNA-cGAS-STING axis is thus a crucial regulator of odontoblast inflammation and could be a potent therapeutic target to prevent severe bacterial inflammation.

## Methods

The Additional file provides primer sequences, antibodies and siRNA sequences.

### Human dental pulp samples

Written informed consent was obtained from the patients at the School and Hospital of Stomatology, Wuhan University, and the study was approved by the Institutional Ethics Board of Wuhan University. In this study, tissue samples were obtained from 18 patients, including 6 healthy patients, 6 carious patients, and 6 pulpitis patients. The healthy teeth group consisted of third molars extracted for orthodontics, without any caries lesions or periodontitis. The carious teeth group consisted of teeth collected from patients diagnosed with caries and carious lesions but without any history of spontaneous pain. The pulpitis group consisted of teeth collected from patients diagnosed with irreversible pulpitis with spontaneous pain, heat- or cold-stimulating pain and pain at night [21, 22].

### Cell culture

The preodontoblast cell line mDPC6T is a cell line that was established by our laboratory [23]. mDPC6T cells were cultured in Dulbecco's modified Eagle medium (DMEM, HyClone) supplemented with 10% fetal bovine serum (FBS, Gibco) at 37 °C in a humidified 5% CO_2_ atmosphere. LPS (Sigma) was added to the medium to establish an LPS-induced odontoblast injury model. Ethidium bromide (EtBr, Thermo Fisher Scientific) was used to deplete mtDNA and was incubated with the cells for 48 h (1 μg/ml) as described.

### RNA preparation and real-time PCR

A total RNA miniprep kit (Axygen) was used to extract total RNA from mDPC6T cells according to the manufacturer's instructions. Real-time PCR was performed as previously described. The primer sequences are listed in the Additional file.

### Protein extraction and western blotting

Total protein samples were collected from mDPC6T cells and tissues using RIPA buffer (Beyotime) containing a phosphatase inhibitor cocktail and protease inhibitor cocktail (MCE). Cytosolic and nuclear protein fractions were isolated by a nuclear and cytoplasmic protein extraction kit (Beyotime). Western blotting was performed according to a previous procedure [24]. The information for the antibodies used is listed in the Additional file.

### RNA interference

siRNAs were purchased from Genepharma (China, Shanghai). mDPC6T cells were transfected with 20 nM siRNA and Lipofectamine 3000 (Invitrogen) according to the manufacturer's protocol. Two days later, mDPC6T cells were transfected with siRNAs again and treated with LPS. Nonspecific siRNA was used as a negative control. The siRNA sequences used in this study are shown in the Additional file.

### Immunocytochemistry

All 3 groups of human tooth samples were demineralized in 10% EDTA buffer for 3 months and then embedded in paraffin. The samples were analyzed according to a previous protocol, and the staining process was performed as previously described [25]. The antibodies used are shown in the Additional file.

### DNA isolation and mtDNA copy number analysis

The cytosol of mDPC6T cells was extracted with the Mitochondria Isolation Kit (Beyotime) according to the manufacturer's protocol. The cytosolic supernatant was collected without mitochondria. The genomic DNA miniprep kit (Axygen) was used to isolate the DNA from the collected cytoplasm supernatant. DNA (10 ng) was subjected to real-time PCR on a CFX96 system (Bio-Rad) with SYBR qPCR master mix (Vazyme). The short regions of the tRNA-Leu^UUR^ and β2-microglobulin genes were amplified to determine mitochondrial DNA and nuclear DNA, respectively, as previously described [26]. The mtDNA/nDNA ratio was calculated to determine the mtDNA copy number. All primers are listed in the Additional file.

### mtDNA isolation and transfection

mtDNA was isolated from mDPC6T cells using a mitochondrial DNA isolation kit (BioVision) according to the protocol. The extracted mtDNA was resuspended in TE buffer and stored at -20 °C for future use. mDPC6T cells were seeded in 6-well plates and transfected with mtDNA (1 μg/well) and Lipofectamine 3000 (Thermo Fisher Scientific) according to the manufacturer’s instructions.

### mtROS measurement

MitoSOX Red dye (Invitrogen; M36008) was used to measure mtROS according to the manufacturer's protocol. mDPC6T cells were stained with 5 μM of MitoSOX Red Dye for 10 min after treated with LPS (20 μg/ml) for 24 h. After staining, cells were washed with PBS and fixed in paraformaldehyde for 10 min. Then, mDPC6T cells were counterstained with DAPI for 10 min. Stained cells were observed and photographed on a confocal microscope (Olympus, Japan).

#### Immunofluorescence analysis

mDPC6T cells were seeded in confocal dishes and treated with LPS (20 μg/ml) for 24 h. Next, mDPC6T cells were incubated with MitoTracker (Invitrogen) using a working concentration of 200 nM for 15 min. After staining, mDPC6T cells were fixed in paraformaldehyde and permeabilized with 0.1% Triton X-100. After 10% bovine serum albumin blocking, mDPC6T were incubated with primary antibodies overnight at 4 ℃. Alexa Fluor 488 secondary antibodies were used for signal detection. Labeled mDPC6T cells were observed with a confocal microscope (Olympus, Japan). The antibodies used are shown in the Additional file.

#### Statistical analysis

Statistical analysis was performed using Student's t-tests or one-way analysis of variance at a significance level of p < 0.05 using GraphPad Prism 8. All data are presented as the means ± standard deviations and are representative of at least three independent experiments. The quantification of western blot results was performed using Fiji software.

## Results

### High expression of cGAS and STING was detected in caries and pulpitis tissues

**Fig. 1:**
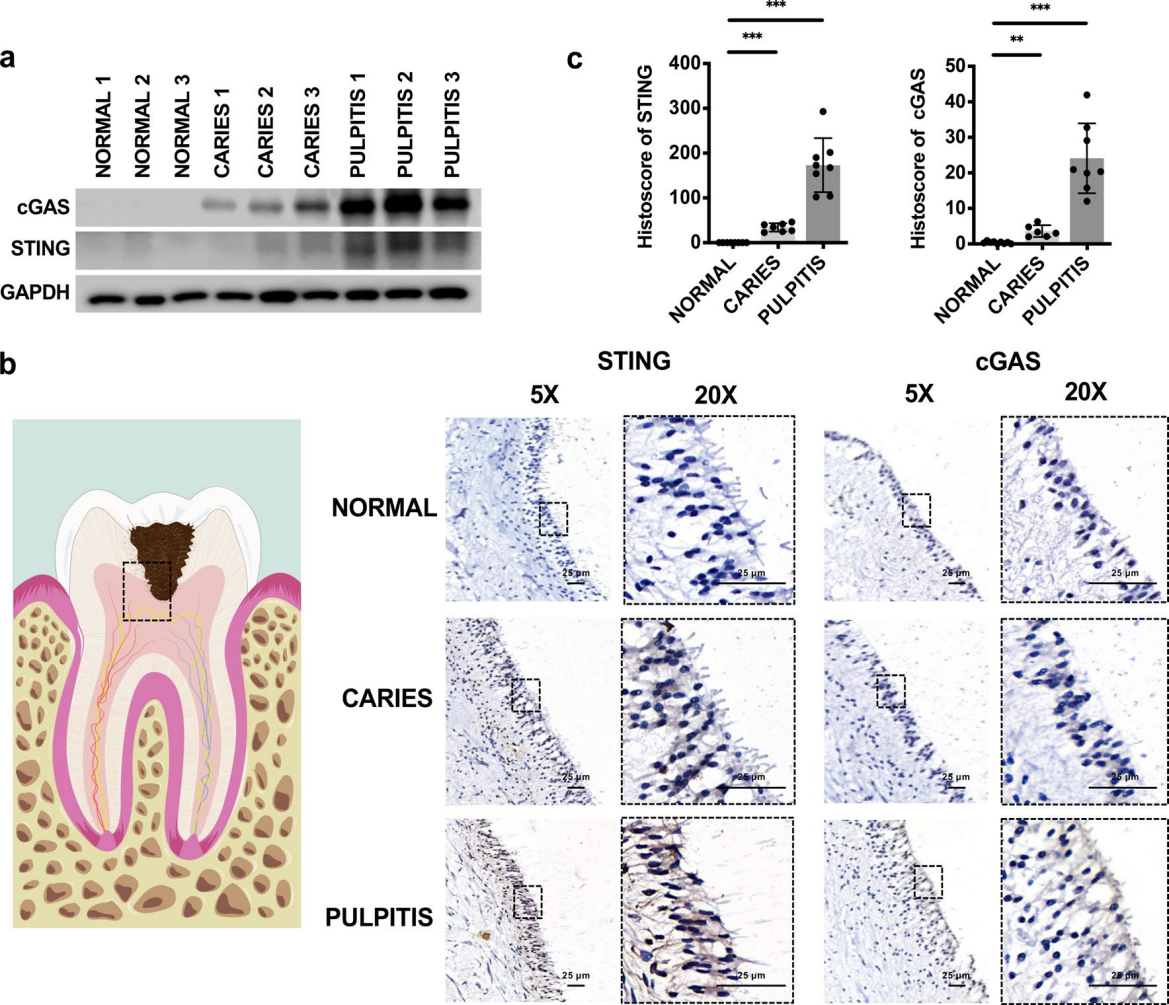
High expression levels of cGAS and STING in inflamed dental pulp. **a** The expression of cGAS and STING in normal tissues, caries tissues and pulpitis tissues was measured by western blotting (n = 3). **b** cGAS and STING expression in the odontoblast layer was visualized as a schematic model (left) by immunohistochemical (IHC) staining (right). Scale bar: 25 μm. **c** Histoscores of cGAS and STING were determined from cGAS and STING expression levels in normal tissues (n = 8), caries tissues (n = 7) and pulpitis tissues (n = 8). The data are the mean ± SEM (*p < 0.05, **p < 0.01 ***, p < 0.001)

To investigate the clinical importance of cGAS and STING in caries and pulpitis patients, we compared cGAS and STING expression levels in caries, pulpitis and normal tooth samples by western blotting (Fig. 1a) and immunohistochemistry (Fig. 1b, c). The western blotting results showed that the expression of cGAS and STING in pulpitis tissues was much higher than that in normal tissues and caries tissues (Fig. 1a). We also found high expression levels of cGAS and STING mainly in the odontoblast layer in caries tooth samples and pulpitis tooth samples compared with healthy tooth samples (Fig. 1b, c). Moreover, the expression of cGAS and STING was significantly increased with progressive exacerbation of inflammation in patient tooth samples (Fig. 1b, c). These data demonstrated that the expression of cGAS and STING positively correlated with inflammation in caries and pulpitis tissues.

### The cGAS-STING pathway was activated by LPS in odontoblast-like cells

**Fig. 2:**
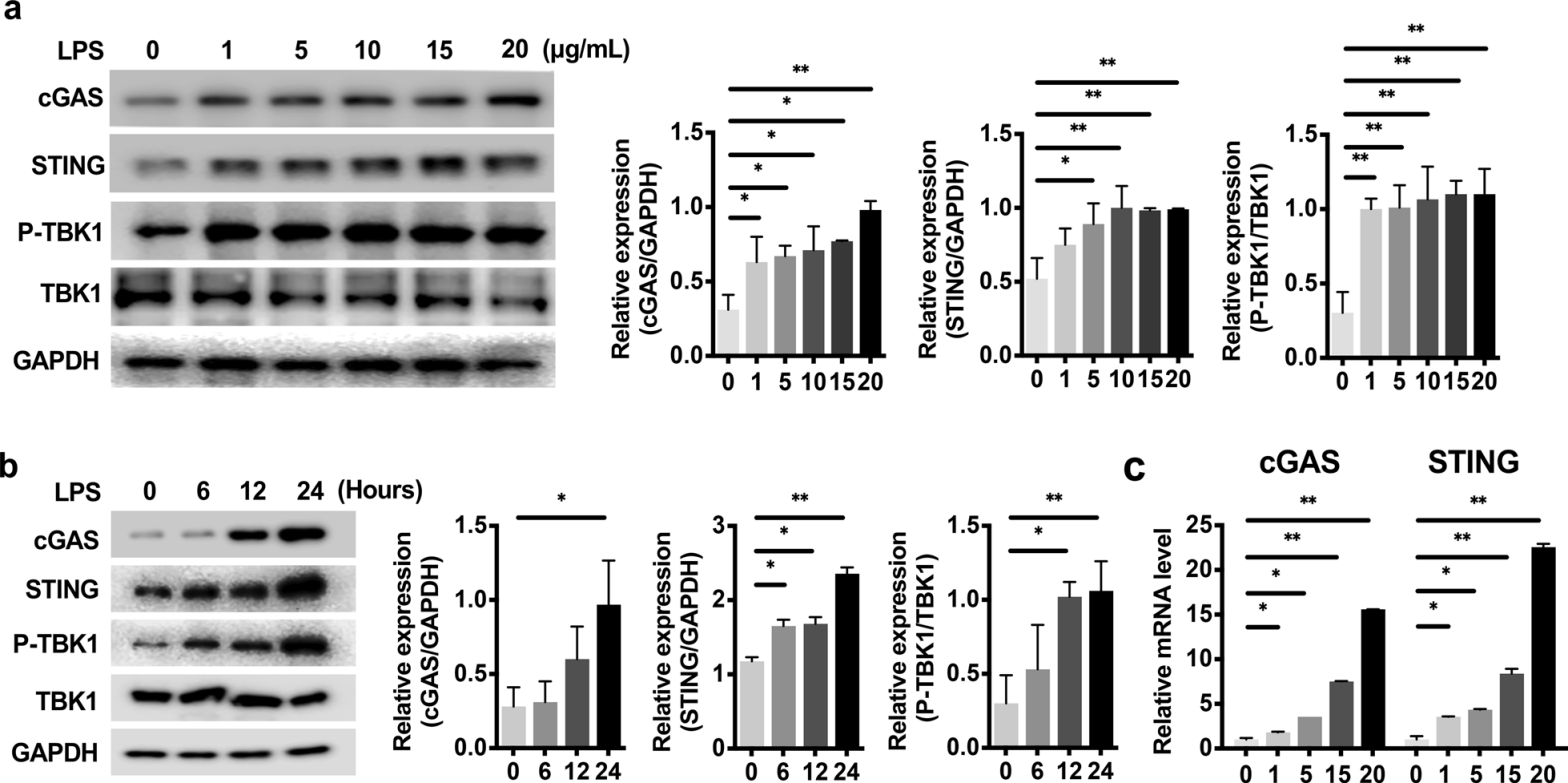
The cGAS-STING pathway was activated by LPS in mDPC6T cells. **a–b** The expression of cGAS and STING and the phosphorylation of TBK1 in mDPC6T cells were assessed by western blotting. mDPC6T cells were treated with different concentrations of LPS (1, 5, 10, 15, and 20 μg/ml) for 24 h (**a**) or 20 μg/ml LPS for 0, 6, 12, and 24 h to induce inflammation (**b**). The quantitative data represent the relative ratio of the target protein to total TBK1 or GAPDH. **c** Real-time PCR was performed to quantify the relative mRNA levels of cGAS and STING in mDPC6T cells after 24 h of stimulation with different concentrations of LPS. The data are the mean ± SEM, n = 3 (*p < 0.05, **p < 0.01 ***p < 0.001)

To further understand the mechanism by which the cGAS-STING pathway promotes odontoblast inflammation, we evaluated changes in the expression of cGAS-STING pathway components in an LPS-stimulated odontoblast-like cell line (mDPC6T) in vitro. The expression of cGAS and STING was upregulated by LPS in mDPC6T cells in a dose-dependent (Fig. 2a) and time-dependent manner (Fig. 2b). mDPC6T cells were stimulated with 20 µg/ml LPS, and cGAS and STING expression was increased after 24 h compared with 6 h and 12 h (Fig. 2b). Real-time PCR confirmed that LPS activated cGAS and STING mRNA expression in mDPC6T cells (Fig. 2c). Consistent with this observation, the phosphorylation of the downstream molecule TBK1 also suggested that the cGAS-STING pathway was activated by LPS (Fig. 2a, b).

### cGAS-STING activation exacerbated LPS-induced mDPC6T inflammation

**Fig. 3:**
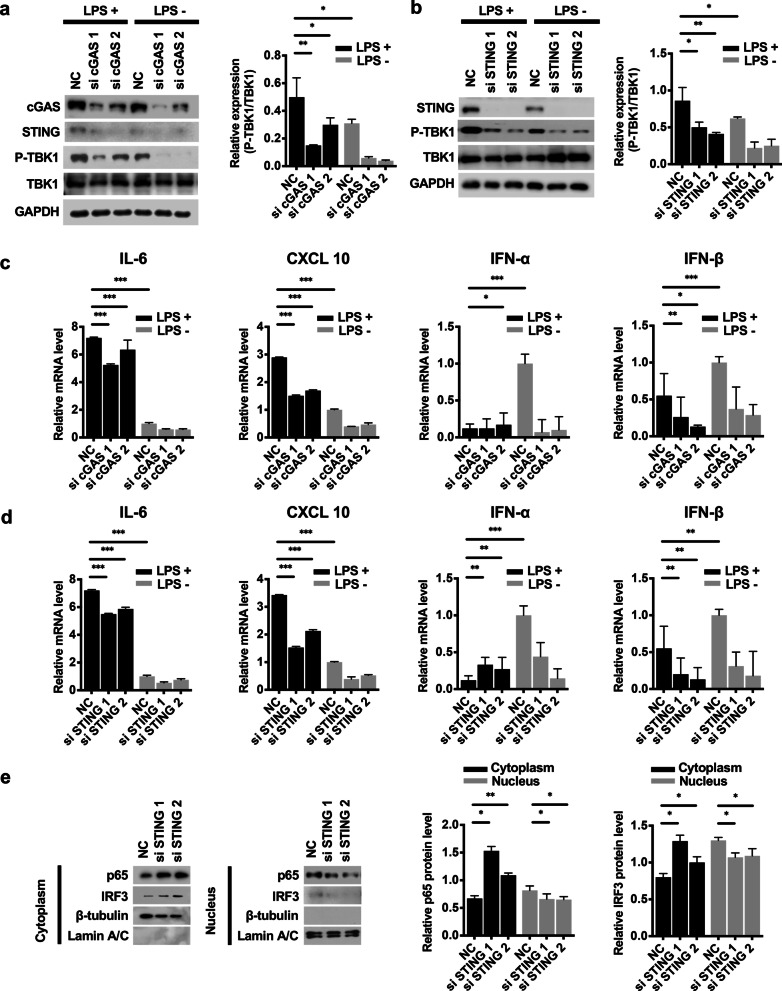
The cGAS-STING pathway regulated LPS-induced mDPC6T inflammation. **a–b** mDPC6T cells were transfected with anti-cGAS siRNA (**a**) or anti-STING siRNA (**b**) twice at an interval of 48 h. LPS (20 μg/ml) was administered 24 h after the second transfection. The phosphorylation of TBK1 was analyzed by western blotting. The quantitative data represent the relative ratio of the target protein to total TBK1. **c–d** Real-time PCR was performed to detect the expression of IL-6, CXCL10, IFN-α and IFN-β in mDPC6T cells in response to 24 h of LPS stimulation without or with cGAS (**c**) or STING (**d**) knockdown. GAPDH served as the loading control. **e** Western blotting was performed to analyze the cytoplasmic and nuclear protein levels of p65 and IRF3 in STING-knockdown mDPC6T cells. β-tubulin served as the loading control for cytoplasmic proteins. Lamin A/C served as the loading control for nuclear proteins. The data are the mean ± SEM, n = 3 (*p < 0.05, **p < 0.01 ***p < 0.001)

To validate the effect of knocking down cGAS or STING on LPS-induced mDPC6T cells, we next analyzed the protein level of the downstream STING target protein TBK1 (Fig. 3a, b) and the mRNA expression level of proinflammatory cytokines (Fig. 3c, d). siRNA-mediated knockdown of cGAS or STING significantly decreased the LPS-induced phosphorylation of TBK1 (Fig. 3a, b). In addition, the increased expression of IL-6 and CXCL10 induced by LPS was significantly reversed by cGAS or STING knockdown (Fig. 3c and d), suggesting that inhibiting cGAS or STING ameliorates odontoblast inflammation. However, IFN-α and β were not induced by LPS treatment after cGAS or STING knockdown (Fig. 3c, d).

Furthermore, we also observed that cGAS or STING affected the nuclear translocation of p65 and IRF3. After knocking down STING in mDPC6T cells, we measured the expression of p65 and IRF3 in the nucleus and cytoplasm (Fig. 3e). The protein levels of p65 and IRF3 in the cytoplasm were increased after STING knockdown (Fig. 3e). Consistently, nuclear p65 and IRF3 protein levels were decreased (Fig. 3e). These results demonstrated that STING knockdown blocked the nuclear translocation of p65 and IRF3.

### LPS induced mitochondrial injury and mtDNA leakage into the cytosol in mDPC6T cells

**Fig. 4:**
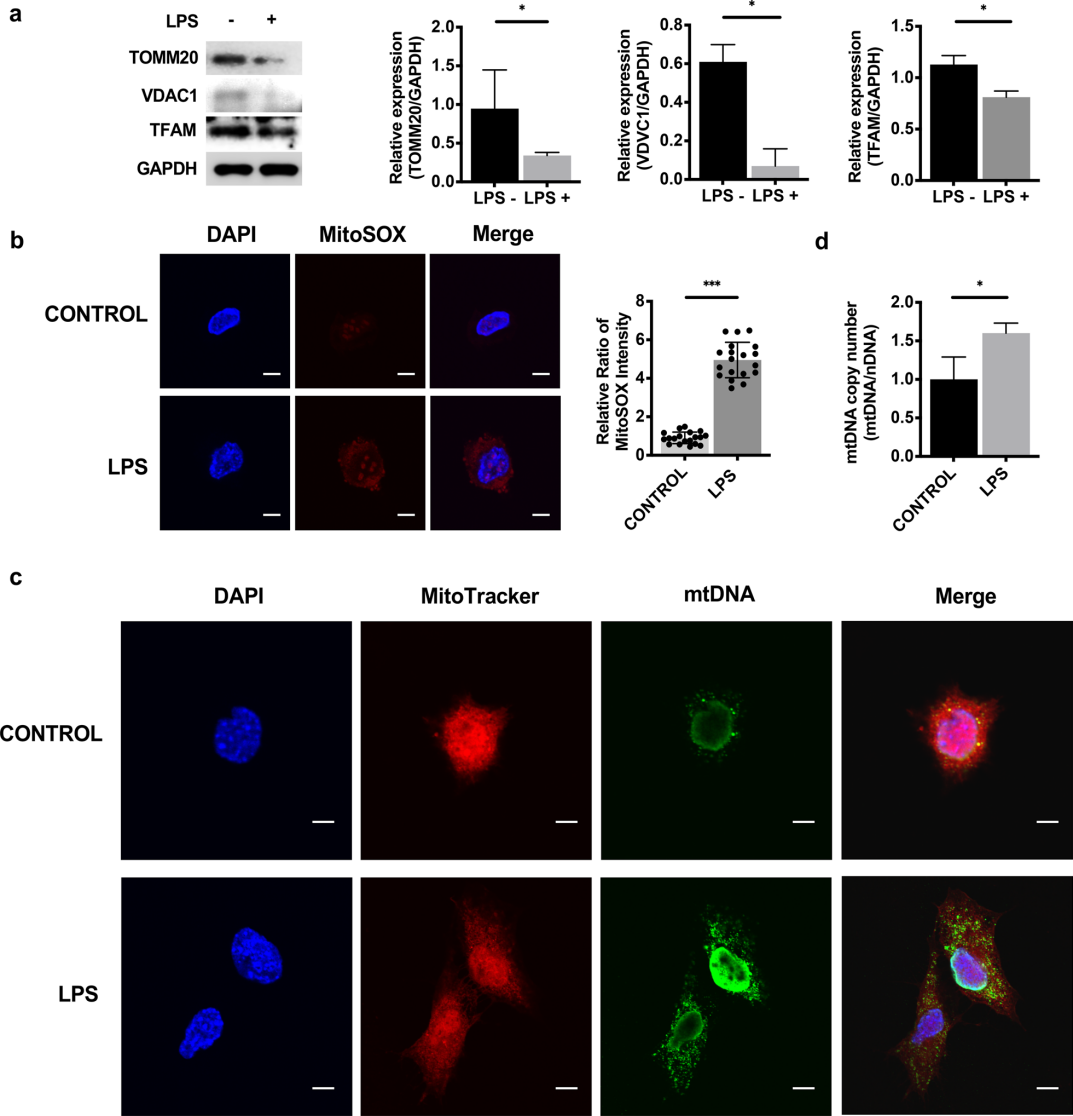
LPS induced mitochondrial injury and mtDNA leakage into the cytosol in mDPC6T cells. **a** Western blotting was performed to assess the expression levels of TOMM20, VDAC1 and TFAM in mDPC6T cells after stimulation with LPS (20 μg/ml, 24 h). **b** Mitochondrial superoxide was evaluated by MitoSOX Red staining in mDPC6T cells after stimulation with LPS (20 μg/ml, 24 h). The box plot shows the signal intensity (n = 20). **c** Immunofluorescent double-labeling of DNA and mitochondria in mDPC6T cells after stimulation with LPS (20 μg/ml) for 24 h was observed by confocal microscopy. Mitochondria was stained with MitoTracker (red). Double-stranded DNA was stained with anti-dsDNA antibodies (green). Scale bar: 10 μm. **d** mtDNA copy number in the cytoplasm was assessed by real-time PCR in mDPC6T cells after LPS stimulation (20 μg/ml, 24 h). The data are the mean ± SEM, n = 3 (*p < 0.05, **p < 0.01 ***p < 0.001)

To elucidate the mechanism of the cGAS-STING pathway in LPS-induced odontoblast inflammation, we focused on the relationship between the cGAS-STING pathway and mitochondrial dysfunction, which may cause mtDNA leakage into the cytosol. We found downregulated expression of the mitochondrial outer membrane proteins TOMM20 and VDAC1 after LPS challenge (Fig. 4a) [27, 28]. Moreover, the expression of the mtDNA-binding protein mitochondrial transcription factor A (TFAM) was also downregulated in inflamed mDPC6T cells (Fig. 4a) [13, 29]. To investigate mitochondrial function in inflamed mDPC6T cells, we evaluated mtROS by MitoSOX Red staining. Mitochondrial function was impaired in inflamed mDPC6T cells, as indicated by the increased production of mtROS (Fig. 4b). In addition, in inflamed mDPC6T cells, intact mtDNA structures were observed by staining with an anti-dsDNA antibody (Fig. 4c). We observed that high levels of dsDNA translocated into the cytosol in inflamed mDPC6T cells and that some dsDNA colocalized with mitochondria (Fig. 4c). To confirm whether mtDNA was released into the cytosol, we quantified cytosolic mtDNA in mDPC6T cells with or without LPS stimulation. We found significant enrichment of cytosolic mtDNA in mDPC6T following LPS challenge (Fig. 4d).

### mtDNA leakage into the cytosol activated the cGAS-STING pathway in LPS-induced mDPC6T cells

**Fig. 5:**
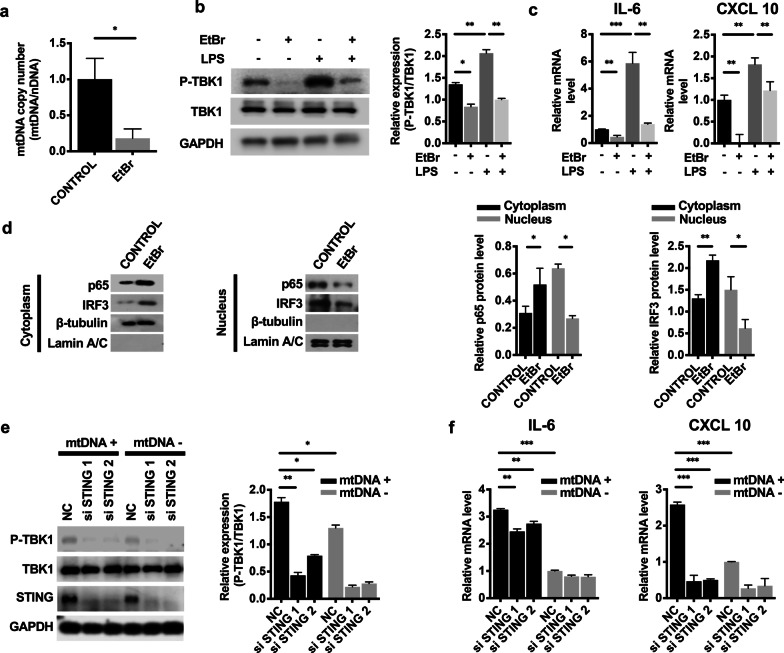
mtDNA leakage into the cytosol activated the cGAS-STING pathway in inflamed mDPC6T cells. **a** mtDNA copy number was assessed by quantitative PCR in mDPC6T cells after treatment with the mtDNA inhibitor EtBr (1 μg/ml, 48 h). **b** Western blotting was used to analyze the phosphorylation of TBK1 in mDPC6T cells after treatment with EtBr (1 μg/ml, 48 h). The quantitative data represent the relative ratio of the target protein to total TBK1. **c** Real-time PCR was performed to measure the expression of IL-6 and CXCL10 in mDPC6T cells after treatment with EtBr. **d** Western blotting was performed to analyze the cytoplasmic and nuclear protein levels of p65 and IRF3 in mDPC6T cells after treatment with EtBr. β-tubulin served as the loading control for cytoplasmic proteins. Lamin A/C served as the loading control for nuclear proteins. **e** mDPC6T cells were cotransfected with anti-STING siRNA and mtDNA (1 μg/ml). Western blotting was used to analyze the phosphorylation of TBK1. The quantitative data represented the relative ratio of the target protein to total TBK1. **f** Real-time PCR was performed to measure the expression of IL-6 and CXCL10 in mDPC6T cells after cotransfection with anti-STING siRNA and isolated mtDNA. The data are the mean ± SEM, n = 3 (*p < 0.05, **p < 0.01, ***p < 0.001)

To determine the role of cytosolic mtDNA in the cGAS-STING pathway, we used EtBr to block the replication of mtDNA but not nuclear DNA [30]. After the application of EtBr, mtDNA was depleted from mDPC6T cells, and the mtDNA copy number was reduced by approximately 80% (Fig. 5a). LPS-induced phosphorylation of TBK1 was restricted in mtDNA-depleted mDPC6T cells (Fig. 5b), and subsequently, the proinflammatory cytokines IL-6 and CXCL10 were markedly attenuated (Fig. 5c). To confirm the role of mtDNA in the nuclear translocation of p65 and IRF3, we analyzed the protein expression of p65 and IRF3 in the nucleus and cytoplasm in mDPC6T cells after treatment with EtBr. Cytosolic p65 and IRF3 levels were increased after EtBr treatment, as analyzed by western blotting (Fig. 5d). Consistently, nuclear p65 and IRF3 levels were decreased (Fig. 5d) compared with those in the control groups. These results showed that mtDNA depletion inhibited the nuclear import of p65 and IRF3 (Fig. 5d).

To confirm the regulatory effect of cytosolic mtDNA on STING in inflamed mDPC6T cells, we isolated mtDNA and transfected it into STING-knockdown mDPC6T cells. The transfected cytosolic mtDNA rescued the phosphorylation of TBK1, which was inhibited by STING knockdown (Fig. 5e), and induced the secretion of the proinflammatory cytokines IL-6 and CXCL10 (Fig. 5f). Release of mtDNA into the cytosol activated STING, resulting in elevated inflammation. In short, these results showed that leaked cytosolic mtDNA plays an important role in inflamed mDPC6T cells via the cGAS-STING pathway.

## Discussion

This study uncovered a novel cellular response to mtDNA that engages the antimicrobial innate immune response in odontoblasts. Our results demonstrated that mitochondrial dysfunction led to mtDNA leakage, which activated the cGAS-STING pathway and the inflammatory cytokines IL-6 and CXCL10 in inflamed mDPC6T cells. Thus, therapeutic strategies targeting this pathway might be an effective way to protect the odontoblast barrier following cariogenic bacterial stimulation.

In our previous study, we found that autophagy was activated to regulate odontoblast differentiation [31] and that Parkin-dependent mitophagy was activated to degrade impaired mitochondria in LPS-induced odontoblasts [21]. Several studies have shown that the accumulation of abnormal mitochondria and cytoplasmic transport of mtDNA are activated by the consumption of autophagic proteins, which depend on the NALP3 inflammasome and mtROS [32]. In our study, we found that the expression of TOMM20 and VDAC1, which are located in the mitochondrial outer membrane and regulate mitochondrial permeability [27, 28], was significantly decreased after LPS stimulation (Fig. 4a). The expression of TFAM, which is the most abundant protein associated with mtDNA and is crucial for maintaining mtDNA structure, transcription, and replication [13, 29], was significantly decreased by LPS stimulation (Fig. 4a). Moreover, we detected mtDNA leakage into the cytosol (Fig. 4c) after stimulation with LPS. To confirm the release of mtDNA, we quantified mtDNA in isolated cytosolic fractions by PCR (Fig. 4d). We found significant enrichment in mtDNA in the cytosol under LPS conditions compared with that in the control groups. These data demonstrated the possible relationship between mtDNA and the odontoblast inflammatory response. Mitochondrial damage was exacerbated by inflammation, which caused mtDNA release into the cytosol.

Various mitochondrial stresses including bacterial infection can lead to mtDNA release into cytosol. Infection with the bacterial pathogen such as *mycobacterium tuberculosis* can trigger cGAS and then stimulate IRF3-dependent type I interferon response. Mitochondrial dynamics and cytosolic mtDNA contribute to IFN-β induction by *mycobacterium tuberculosis* [33]. H_2_O_2_ induced by *Streptococcus pneumoniae* resulted in mitochondrial damage, mtDNA leakage and IFN type I production in alveolar epithelia cells [34]. Pathogen-infected cells also secrete IL-1β activating the cGAS-STING pathway in surrounding bystander cells. IL-1β stimulation of bystander cells promotes mitochondrial dysfunction, decreases mitochondrial membrane potential and induces mtDNA leakage into cytosol [35–37].

Emerging evidence suggests that mtDNA is a major inflammatory activator when it leaks from stressed mitochondria and can promote the progression of various inflammation-related diseases [11, 15, 16, 38]. In our study, we found that the depletion of mtDNA could restrict the secretion of the inflammatory cytokines IL-6 and CXCL10. These results provide evidence of a link between mtDNA and the odontoblast inflammatory response. This result suggested that mtDNA, as an important inflammatory driver, could participate in cellular mechanisms that contribute to the progression of odontoblast inflammation and affect the dentin-pulp complex that defends against microbial invasion.

Emerging evidence has shown that mtDNA acts as a cell-intrinsic cGAS ligand to trigger the STING signaling pathway in certain contexts to drive inflammation [7, 15, 18]. In our study, after utilizing the mtDNA inhibitor EtBr, we found that phosphorylation of the STING pathway downstream mediators TBK1 was inhibited (Fig. 5b). mtDNA rescued the activation of TBK1 phosphorylation after STING knockdown (Fig. 5e). Depletion of mtDNA inhibited the nuclear translocation of p65 and IRF3 (Fig. 5d) and reduced the expression of IL-6 and CXCL10 (Fig. 5c). Therefore, our findings strongly suggested that mtDNA promoted odontoblast inflammation by activating cGAS-STING signaling and inducing inflammatory cytokine release. The mechanism by which mtDNA regulates the cGAS-STING pathway in inflamed odontoblasts in our study was consistent with that of a previous study in human tubular epithelial cells [39]. The role of the mtDNA-cGAS-STING axis in an animal model of pulpitis requires further investigation.

## Conclusions

Our study revealed a previously unreported mechanism of the cellular immune response through the mtDNA-cGAS-STING axis in odontoblast inflammation. These data suggest that therapeutic STING inhibitors may be used as anti-inflammatory drugs for pulpitis treatment.

## Data Availability

Not applicable.

## Abbreviations

mtDNA: Mitochondrial DNA
cGAS: Cyclic GMP-AMP synthase
STING: Stimulator of interferon genes
EtBr: Ethidium bromide
TBK1: TANK-binding kinase 1
NF-κB: Nuclear factor kappa-B
IRF3: Interferon regulatory factor 3
nDNA: Nuclear DNA
PRRs: Pattern recognition receptors
mtROS: Mitochondrial reactive oxygen species
mtDAMPs: Mitochondrial danger-associated molecular patterns
TFAM: Mitochondrial transcription factor A
LPS: Lipopolysaccharide
